# Comparing infectivity and virulence of emerging SARS-CoV-2 variants in Syrian hamsters

**DOI:** 10.1016/j.ebiom.2021.103403

**Published:** 2021-05-25

**Authors:** Rana Abdelnabi, Robbert Boudewijns, Caroline S. Foo, Laura Seldeslachts, Lorena Sanchez-Felipe, Xin Zhang, Leen Delang, Piet Maes, Suzanne J.F. Kaptein, Birgit Weynand, Greetje Vande Velde, Johan Neyts, Kai Dallmeier

**Affiliations:** a KU Leuven Department of Microbiology, Immunology and Transplantation, Rega Institute for Medical Research, Laboratory of Virology and Chemotherapy, B-3000 Leuven, Belgium; b Molecular Vaccinology and Vaccine Discovery, Belgium; c KU Leuven Department of Imaging and Pathology, Biomedical MRI and MoSAIC, 3000, Leuven, Belgium; d Laboratory of Clinical and Epidemiological Virology, Rega Institute, KU Leuven, Department of Microbiology, Immunology and Transplantation, 3000, Leuven, Belgium; e Zoonotic Infectious Diseases Unit, Leuven, Belgium; f KU Leuven Department of Imaging and Pathology, Translational Cell and Tissue Research, B-3000 Leuven, Belgium; g GVN, Global Virus Network, Baltimore, MD, USA

**Keywords:** Emergence, Hamster model, SARS-CoV-2, Variants of concern (VoC)

## Abstract

**Background:**

Within one year after its emergence, more than 108 million people acquired SARS-CoV-2 and almost 2·4 million succumbed to COVID-19. New SARS-CoV-2 variants of concern (VoC) are emerging all over the world, with the threat of being more readily transmitted, being more virulent, or escaping naturally acquired and vaccine-induced immunity. At least three major prototypic VoC have been identified, i.e. the United Kingdom, UK (B.1.1.7), South African (B.1.351) and Brazilian (B.1.1.28.1) variants. These are replacing formerly dominant strains and sparking new COVID-19 epidemics.

**Methods:**

We studied the effect of infection with prototypic VoC from both B.1.1.7 and B.1.351 variants in female Syrian golden hamsters to assess their relative infectivity and virulence in direct comparison to two basal SARS-CoV-2 strains isolated in early 2020.

**Findings:**

A very efficient infection of the lower respiratory tract of hamsters by these VoC is observed. In line with clinical evidence from patients infected with these VoC, no major differences in disease outcome were observed as compared to the original strains as was quantified by (*i*) histological scoring, (*ii*) micro-computed tomography, and (*iii*) analysis of the expression profiles of selected antiviral and pro-inflammatory cytokine genes. Noteworthy however, in hamsters infected with VoC B.1.1.7, a particularly strong elevation of proinflammatory cytokines was detected.

**Interpretation:**

We established relevant preclinical infection models that will be pivotal to assess the efficacy of current and future vaccine(s) (candidates) as well as therapeutics (small molecules and antibodies) against two important SARS-CoV-2 VoC.

**Funding:**

Stated in the acknowledgment.

**Research in Context**

**Evidence before this study**

New SARS-CoV-2 variants are emerging and in circulation globally, with the threat of being more readily transmitted, more virulent, and/or escaping naturally acquired and vaccine-induced immunity. At least three major prototypic variants have been identified, the United Kingdom, UK (B.1.1.7), South African (B.1.351), and Brazilian (B.1.1.28.1) variants, and these are replacing formerly dominant strains, resulting in new COVID-19 epidemics.

**Added value of this study**

This study investigates the relative infectivity and virulence of prototypic VoC B.1.1.7 and B.1.351 in comparison with two basal SARS-CoV-2 strains in Syrian golden hamsters, a robust model of COVID-19 infection. The findings here indicate that VoC establish a very efficient infection of the lower respiratory tract of hamsters, comparable to that of the basal lineages. Additionally, disease outcome (as quantified by histological scoring, micro-computed tomography, and expression profiles of selected antiviral and pro-inflammatory cytokine genes) was similar to that caused by basal lineages, in line with clinical evidence from patients infected with these VoC. Notably, a particularly strong elevation of pro-inflammatory cytokines was detected in hamsters infected with VoC B.1.1.7.

**Implications of all the available evidence**

This study lays the foundation for relevant pre-clinical infection models, which are necessary to assess current and future vaccines, vaccine candidates, and therapeutics against two important SARS-CoV-2 VoC.

Alt-text: Unlabelled box

## 1 Introduction

Barely 1 year after surfacing and global spread of SARS-CoV-2, more than 113 Mio infected people and 2·5 Million fatal cases have been reported worldwide (as of February 26^th^ 2021). Variants of SARS-CoV-2 are emerging in different parts of the world, posing a new threat. Currently at least three major prototypic virus variants of concern (VoC) have been detected respectively in the United Kingdom, UK (lineage B.1.1.7; [1], [2], [3] earliest sample date 2020-02-03), South Africa (B.1.351 or 501Y.V2; [1,4] earliest sample date 2020-10-08) and Brazil (B.1.1.28.1 or P.1; [5,6] earliest sample date 2020-12-15). Even in highly endemic regions, these VoC started to replace formerly dominant strains and appear to be at the root of new waves of infections and new spikes in excess mortality. In general, numerous mutations have been identified in the VoC genomes, which occur in different coronavirus genes. Though, the Spike (S) protein appears to be particularly prone to accumulate amino acid changes. Notably, mutations found in the SARS-CoV-2 spike protein that may be associated with (*i*) more efficient human-to-human transmission, (*ii*) increased virulence, [7] and/or (*iii*) escape from naturally acquired [6] or vaccine-induced [8,9] immunity raise concerns. The role of such mutations have been experimentally addressed, mostly using synthetic viruses generated by targeted mutagenesis. As such the VoC of UK, South African and Northern Brazil origin share the prominent mutation N501Y in the receptor-binding domain (RBD) of S that may favor viral attachment to its cellular receptor hACE2 [10]. Moreover, massive outbreaks in previously heavily affected regions, such as in the Manaus area of Northern Brazil with seropositivity rates of up to 76% prior to disease resurgence, raise the concern of VoC escaping pre-existing immunity [6]. Likewise, several vaccine candidates either failed to show efficacy, or at least displayed a marked drop in vaccine efficacy in Phase 3 clinical trials in regions of South Africa where the VoC B.1.351 is circulating [8,9]. Escape from antibody neutralization has been linked to amino acid changes in key residues of S, such as E484K, found in both the South African and Brazilian variants and as well in some more recently emerging UK (sub)variants [11], [12], [13]. The evolution of such new SARS-CoV-2 VoC may be driven by viral escape under host immune pressure during acute infection, [14] and resulting SARS-CoV-2 antigenic drift is feared to spark future COVID-19 epidemics.

Here, we investigate infection of Syrian hamsters with prototypic VoC, namely local Belgian low-passage isolates from both B.1.1.7 and B.1.351 variants [15], [16], [17].

Syrian hamsters are a relevant small animal model to study the infectivity and virulence of clinical SARS-CoV-2 isolates [15,[18], [19], [20]]. We show that such VoC (isolated in 2021) efficiently infect the lower respiratory tract of hamsters following intranasal inoculation, resulting in a pathology that is very similar to that observed after infection with SARS-CoV-2 from earlier more basal variants (originating from early 2020). In line with clinical evidence from patients infected with VoC B.1.1.7 and B.1.351, major differences in disease outcome with infections caused with the early 2020 isolates were not observed. Nonetheless, animals infected with VoC B.1.1.7 presented with a particularly elevated expression of proinflammatory cytokines, yet with no obvious impact on further aggravation of lung pathology. Overall, we demonstrate that hamsters can serve as relevant preclinical model that can be used to assess the infectivity of clinical SARS-CoV-2 isolates including that of current VoC. This model will also serve as an important tool to study the efficacy of current and future vaccines [17,21,22] and therapies [16,23].

## 2 Methods

### 2.1 Virus isolation and virus stocks

All virus-related work was conducted in the high-containment BSL3 facilities of the KU Leuven Rega Institute (3CAPS) under licenses AMV 30112018 SBB 219 2018 0892 and AMV 23102017 SBB 219 2017 0589 according to institutional guidelines.

**Fig. 1:**
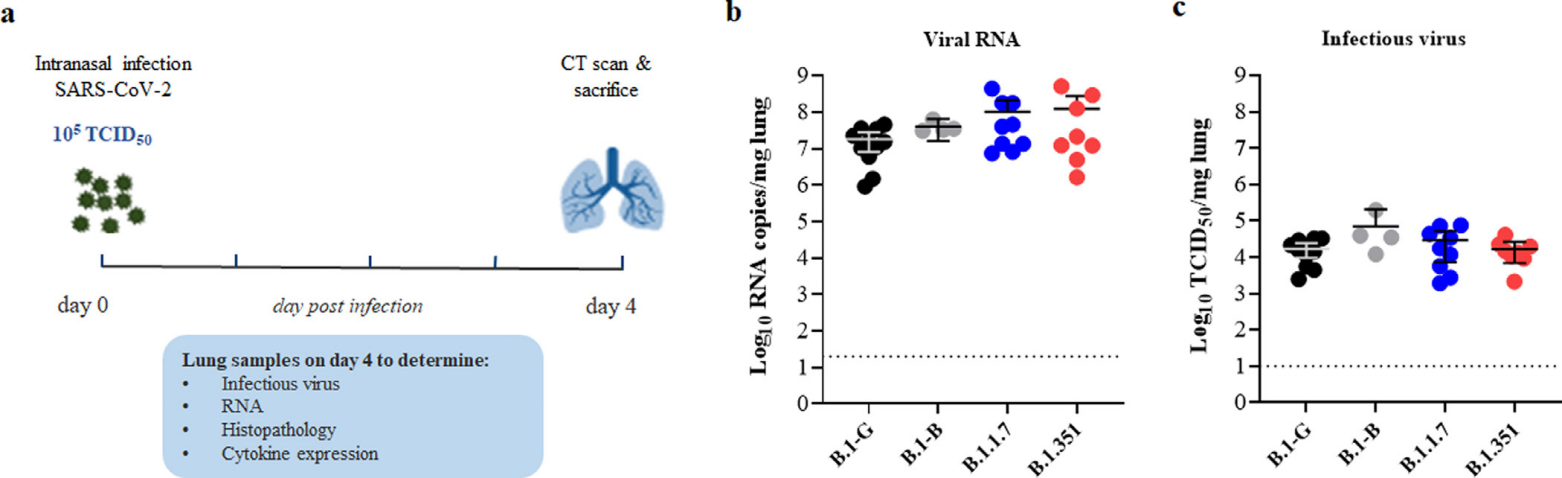
**Viral loads from the lungs of Syrian hamsters infected with different SARS-CoV-2 variants**. (a) Set-up of the Syrian hamster infection study. (b) Viral RNA levels in the lungs of hamsters infected with 10^5^ TCID_50_ of B.1-G (n = 11), B.1-B (n = 4), B.1.1.7 (n = 9) or B.1.351 (n = 8) SARS-CoV-2 variants on day 4 post-infection (pi) are expressed as log_10_ SARS-CoV-2 RNA copies per mg lung tissue. Individual data and mean values with 95% CI are presented. (c) Infectious viral loads in the lungs of hamsters infected with the different SARS-CoV-2 variants at day 4 pi are expressed as log_10_ TCID_50_ per mg lung tissue. Individual data and mean values with 95% CI are presented. All data are from at least two independent experiments except for the B.1-B group.

The basal Severe Acute Respiratory Syndrome-related Coronavirus 2 (SARS-CoV-2) strains isolated in early 2020, i.e. strain BetaCov/Belgium/GHB-03021/2020 (EPI_ISL_407976; 2020-02-03) [24] and strain Germany/BavPat1/2020 (also referred to as BavPat-1, EPI_ISL_406862; 2020-01-28) [25] for simplicity called B.1-G and B.1-B, respectively, have been described elsewhere. B.1-G is most closely related to the prototypic Wuhan-Hu-1 2019-nCoV (GenBank accession number MN908947.3) strain; [15] B.1-B contains the secondary D614G Spike mutation (Fig. 1A). B.1-B was a generous gift of Prof. Christian Drosten, Department of Virology, University Hospital Charité, Berlin, Germany.

SARS-CoV-2 strains belonging to the VoC UK and South African variants B.1.1.7 (hCoV-19/Belgium/rega-12211513/2020; EPI_ISL_791333, 2020-12-21) and B.1.351 (hCoV-19/Belgium/rega-1920/2021; EPI_ISL_896474, 2021-01-11) were each isolated from nasopharyngeal swabs taken from travelers returning to Belgium in December 2020 and January 2021, respectively; B.1.1.7 from a healthy subject and B.1.351 from a patient with respiratory symptoms. All strains B.1-G, B.1.1.7 and B.1.351 isolated in house were subjected to sequencing on a MinION platform (Oxford Nanopore) [26] directly from the nasopharyngeal swabs.

All virus stocks were grown on Vero E6 cells and median tissue culture infectious doses (TCID_50_) defined by titration using the Spearman-Kärber method as described [15]. Virus stocks used throughout this study were from early passages (p); B.1-G and B.1-B from p3, and B.1.1.7 and B.1.351 from p2. All virus stocks showed a homogenous small plaque phenotype (Fig. S1b). Respective Spike gene sequences were confirmed by Sanger sequencing of RT-PCR amplicons of (*i*) virus RNA isolated from virus stocks prior to inoculation and (*ii*) virus RNA isolated from infected hamster lungs 4 dpi. Deep sequencing confirmed a homogenous genetic structure of the early passage virus stocks regarding their Spike sequences; with the majority of genomes in each virus stock 100% identical to the respective original patient isolate and consensus sequence. All amino acid variants thus observed in the Spike protein sequences of the virus strains under study are summarized in Fig. S1a. However, as detected by deep sequencing, the B.1-G (p3) stock harbored some minority Spike sequences variants originating from cell-culture adaptation and resulting in some modifications at the S1/S2 furin cleavage site [15,27]. In case of the B.1-G (p3), this resulted in a five amino acid deletion upstream of the S1/S2 furin cleavage site as described [16]. In the B.1.351 (p2) virus stock about half of the sequence reads revealed a single nucleotide change in the first arginine codon of the poly-arginine stretch of the S1/S2 site resulting in a tryptophan codon without any changes related to the passaging on Vero cells. In case of the B.1.1.7 (p2) and the B.1-B (p2) virus stocks, no minority Spike variants were detectable by deep sequencing.

### 2.2 Infection of hamsters

Housing and experimental infections of hamsters have been described [15], [16], [17]. In brief, 6 to 8 weeks old female Syrian hamsters (*Mesocricetus auratus*) were sourced from Janvier Laboratories and kept per two in individually ventilated isolator cages (IsoCage N Bio-containment System, Tecniplast) at 21 °C, 55% humidity and 12:12 day/night cycles. Animals had ad libitum access to food and water, and a wooden gnawing block and extra bedding material was provided as cage enrichment. All hamsters had SPF status at arrival and manipulations were performed under a laminar flow cabinet. Animals were anesthetized with ketamine/xylazine/atropine and intranasally infected with 50 µL of virus stock containing approximately 1 × 10^5^ TCID_50_ (25 µL in each nostril), and euthanized 4 days post infection (dpi) for sampling of the lungs and further analysis by i.p. injection of 500 μL Dolethal (200 mg/mL sodium pentobarbital).

### 2.3 Study design

A total of 55 animals was used in the study [10^5^ TCID_50_ B.1-G (n = 11); 10^4^ TCID_50_ B.1-G (n = 4); 10^5^ TCID_50_ B.1-B (n = 4); 10^5^ TCID_50_ B.1.1.7 (n = 9); 10^4^ TCID_50_ B.1.1.7 (n = 6); 10^5^ TCID_50_ B.1.351 (n = 8); 10^4^ TCID_50_ B.1.351 (n = 6); 10^2^ TCID_50_ B.1.351 (n = 3); uninfected (n = 4)]. Uninfected hamsters were left untreated. Predefined humane endpoints (20% weight loss, immobility, neurological signs) and two subsequently failed micro-CT scans (motion artifacts) to protect animals from harmful radiation were set as exclusion criteria. No animals had to be completely excluded from the study, yet micro-CT scans of two animals were excluded because of motion artifacts (one animal each in B.1-G and B.1.351 groups). Within a cage, only one virus variant was used to avoid cross-contamination among cage mates. No randomization methods were used and confounders were not controlled, though all caretakers and technicians were blinded to group allocation in the animal facility, and to sample numbers for analysis (qPCR, titration, histology, micro-CT). For summary of the animal study design, a full ARRIVE (Animal Research: Reporting of In Vivo Experiments; https://arriveguidelines.org) list has been added as extra Supplementary Data file.

### 2.4 Sample size justification

Sample sizes maximized considering limits in BSL3 housing capacity, numbers of animals that can be handled under BSL3 conditions, and availability of virus stock (limiting for B.1-B).

### 2.5 Virological and cytokine expression analysis

Virus loads were determined by end-point titration (TCID_50_) and RT-qPCR from lung homogenates [B.1-G (n = 11); B.1-B (n = 4); B.1.1.7 (n = 9); B.1.351 (n = 8)] exactly as described in detail before [15], [16], [17]. RT-qPCR was performed on a LightCycler96 platform (Roche) using the iTaq Universal Probes One-Step RT-qPCR kit (BioRad) with N2 primers and probes targeting the nucleocapsid. Analysis of differential expression of hamster interleukin (IL)-6, IL-10, interferon (IFN)-γ, IFN-λ, IP-10, TNF-α, MX-2 and ACE2 by quantitative RT-qPCR [uninfected controls (n = 4); B.1-G (10^5^ TCID_50_, n = 11); B.1-B (10^5^ TCID_50_, n = 4); B.1.1.7 (10^5^ TCID_50_, n = 9; 10^4^ TCID_50_, n = 6); B.1.351 (10^5^ TCID_50_, n = 8; 10^2^ TCID_50_, n = 3)] has been described [17].

### 2.6 Micro-CT and image analysis

Micro-CT data for the lungs of free-breathing hamsters were acquired on a Skyscan 1278 system (Bruker Belgium) and analyzed as described before [15], [16], [17]. In brief, hamsters were scanned in supine position under isoflurane anesthesia producing expiratory weighted three-dimensional data sets with 50-μm isotropic reconstructed voxel size [28] in approximately 3 min scanning time.

Visualization and quantification of reconstructed micro-CT data were performed with as primary outcome measure a semiquantitative scoring of micro-CT data [28]. Visual observations were blindly scored on five different, predefined transversal tomographic sections for both lung and airway disease by two independent observers, averaged, and scores for the five sections summed up to obtain a score from 0 to 10, reflecting severity of lung and airway abnormalities compared to healthy control hamsters. As secondary measure, imaging-derived biomarkers (non-aerated, aerated and total lung volume) were quantified as before [[15], [16], [17],^29^]. In total, 32 animals were scanned [B.1-G (n = 11); B.1-B (n = 4); B.1.1.7 (n = 9); B.1.351 (n = 8)], yet for two animals no lung scores and/or (non)-aerated lung volumes could be quantified because of motion artifacts (one animal each in B.1-G and B.1.351 groups).

### 2.7 Pathology assessment by histology

Assessment of lung pathology was performed as before [15], [16], [17]. In brief, for histological examination, lungs were fixed overnight in 4% formaldehyde, embedded in paraffin and tissue sections (5 μm) after staining with H&E scored blindly for lung damage, using a cumulative score of 1 to 3 each for an extended range of parameters as recently suggested; [30] i.e. congestion, intra-alveolar hemorrhage, apoptotic bodies in bronchial epithelium, necrotizing bronchiolitis, perivascular edema, bronchopneumonia, perivascular inflammation, peribronchial inflammation, vasculitis, endothelialitis, perivascular cuffs, intraluminal PMN, intraalveolar edema, mesothelial hyperplasia, fibrosis and inflammation. To avoid any sampling bias resulting from inhomogeneous distribution of SARS-CoV-2 induced lesions as frequently observed in Syrian hamsters, [30] from each animal an entire lung was examined, cut in two halves along the long axis of the lung. Both halves were examined, and findings added up for final scoring. Number of hamsters per group: B.1-G (n = 11), B.1-B (n = 4), B.1.1.7 (n = 9) and B.1.351 (n = 8).

### 2.8 Statistical analysis

The number of animals and independent experiments that were performed is indicated in the legends to figures. The analysis of histopathology and CT scan images was done blindly. All statistical analyses were performed using GraphPad Prism 9 software (GraphPad, San Diego, CA, USA). Results are presented as means and 95% confidence intervals. Statistical differences between groups were analyzed using Kruskal-Wallis with Dunn's multiple comparisons test, and considered statistically significant at p-values <0·05 (* p < 0·05, ** p < 0·01, *** p < 0·001). P-values for statistical differences between conditions (Kruskal-Wallis with Dunn's multiple comparisons test) can be found in Supplementary tables S1 and S3.

### 2.9 Ethics

Housing conditions and experimental procedures were done with the approval and under the guidelines of the ethics committee of animal experimentation of KU Leuven (license P065-2020).

### 2.10 Role of funding source

The Funders had no role in study design, data collection, data analyses, interpretation, or writing of report.

### 2.11 Data handling

Data were handled according to Institutional guidelines, following established Data Management Plans endorsed by the KU Leuven iBiosafety Department and animal welfare body, guided by the KU Leuven Internal Research Commission (DOC). Appropriate sample storage and identification is ensured by a database system (FreezerPro) using barcoded labels where appropriate. Collection of primary numerical data was performed using standard software (MS Excel) and electronic lab books (MS OneNote) stored and back-uped on KU Leuven hosted secured servers. Data were unblinded and further analyzed using appropriate statistics software (GraphPad).

## 3 Results

**Fig. 2:**
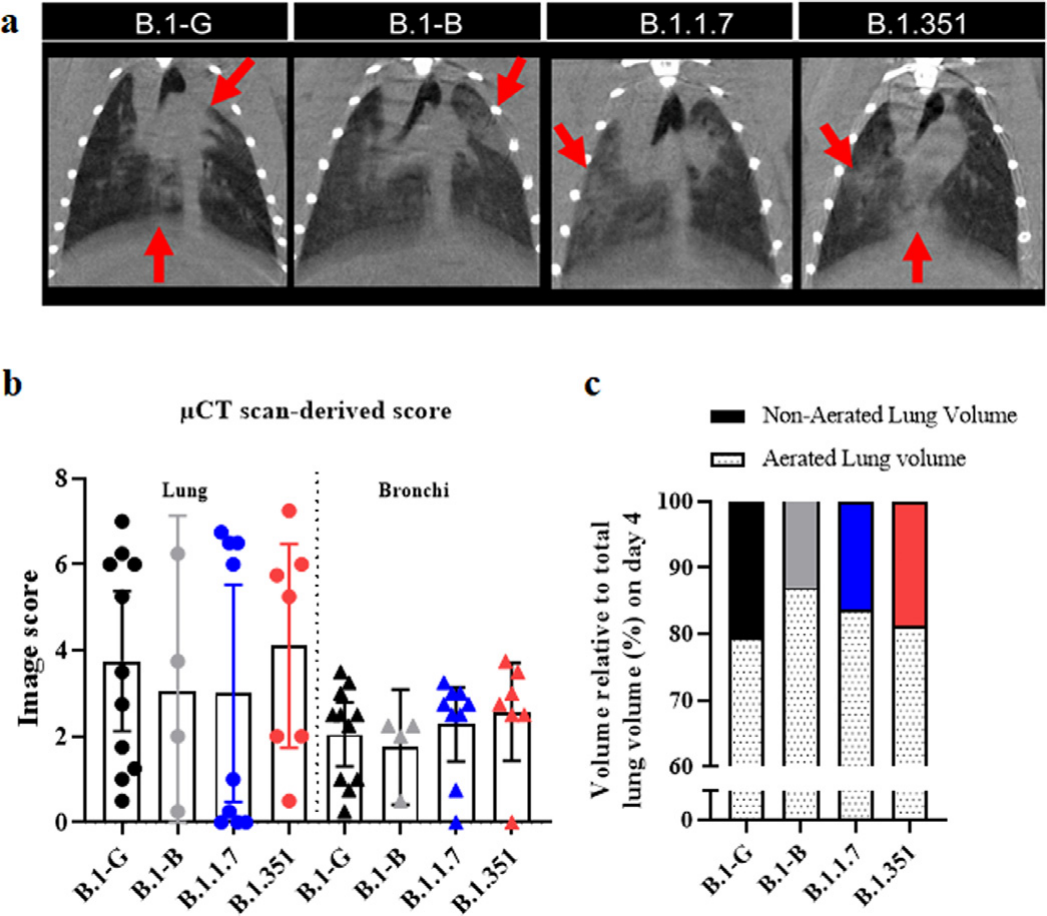
**Micro-CT analysis of lung disease burden in Syrian hamsters infected with different SARS-CoV-2 variants**. (a) Representative coronal lung micro-CT images of hamsters infected with B.1-G, B.1-B, B.1.1.7 or B.1.351 SARS-CoV-2 variants at day 4 pi. Red arrows indicate examples of pulmonary infiltrates. (b) Quantification of the micro-CT-derived lung and bronchi disease scores in hamsters infected with 10^5^ TCID_50_ of B.1-G (n = 10), B.1-B (n = 4), B.1.1.7 (n = 9) or B.1.351 (n = 7) SARS-CoV-2 variants on day 4 post-infection. Individual data per hamster are shown, bars represent mean values with 95% CI. (c) Micro-CT-derived non-aerated lung volume (reflecting the tissue lesion volume) and aerated lung volume relative to total lung volume of hamsters infected with the different SARS-CoV-2 variants. All data are from at least two independent experiments except for the B.1-B group. (For interpretation of the references to color in this figure legend, the reader is referred to the web version of this article.)

**Fig. 3:**
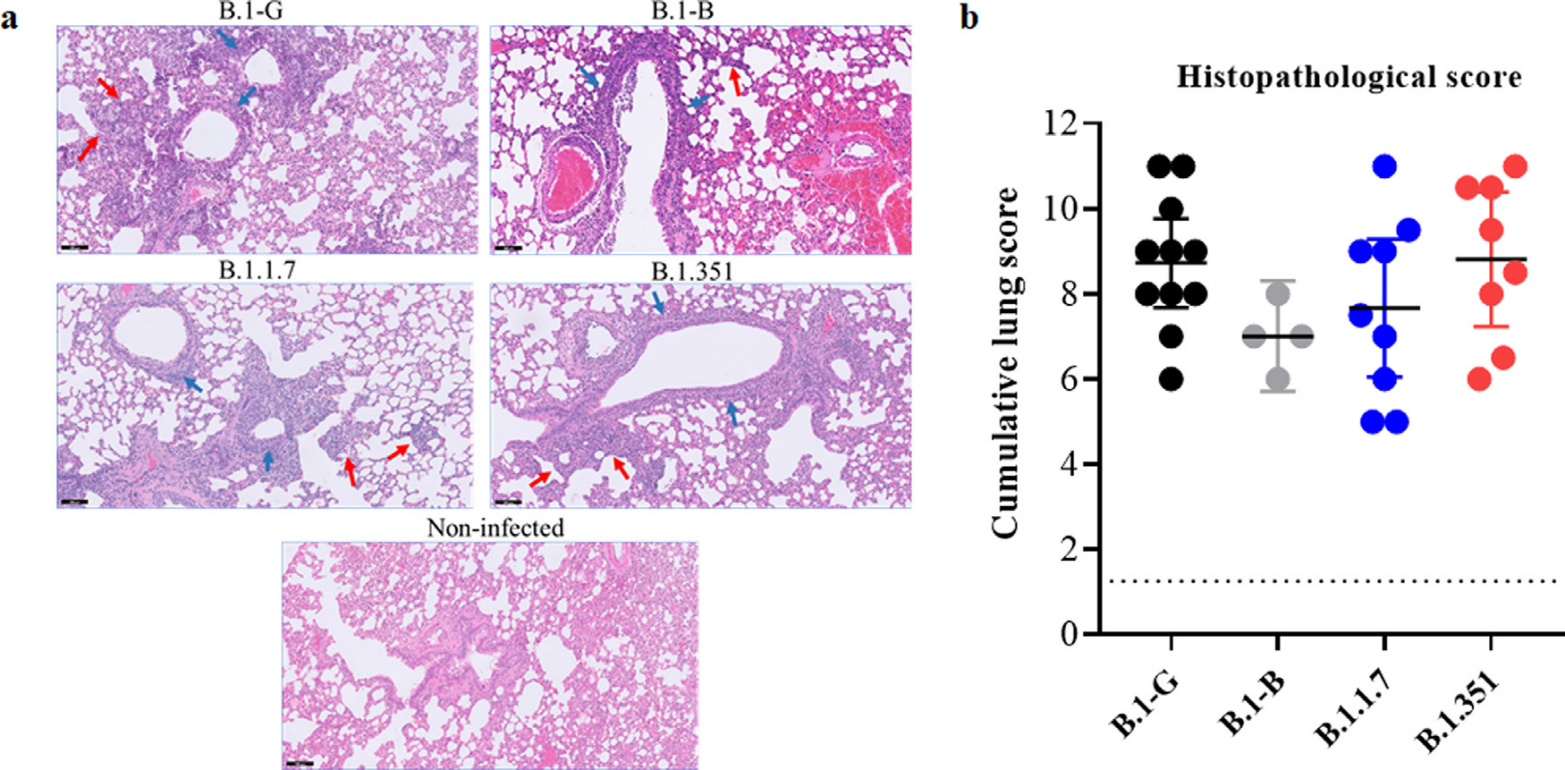
**Histopathology of lungs of Syrian hamsters infected with different SARS-CoV-2 variants**. (a) Representative H&E images of lungs of hamsters infected with 10^5^ TCID_50_ of B.1-G (n = 11), B.1-B (n = 4), B.1.1.7 (n = 9) or B.1.351 (n = 8) SARS-CoV-2 variants at day 4 pi. The lungs of hamsters infected with any of the SARS-CoV-2 variants show peri-bronchial inflammation (blue arrows) and bronchopneumonia in the surrounding alveoli (red arrows), whereas the lungs of non-infected hamster appear normal. Scale bars, 100 μm. (b) Cumulative severity score from H&E stained slides of lungs from hamsters infected with the different SARS-CoV-2 variants. Individual data and mean values with 95% CI are presented and the dotted line represents the median score of untreated non-infected hamsters. All data are from at least two independent experiments except for the B.1-B group. (For interpretation of the references to color in this figure legend, the reader is referred to the web version of this article.)

**Fig. 4:**
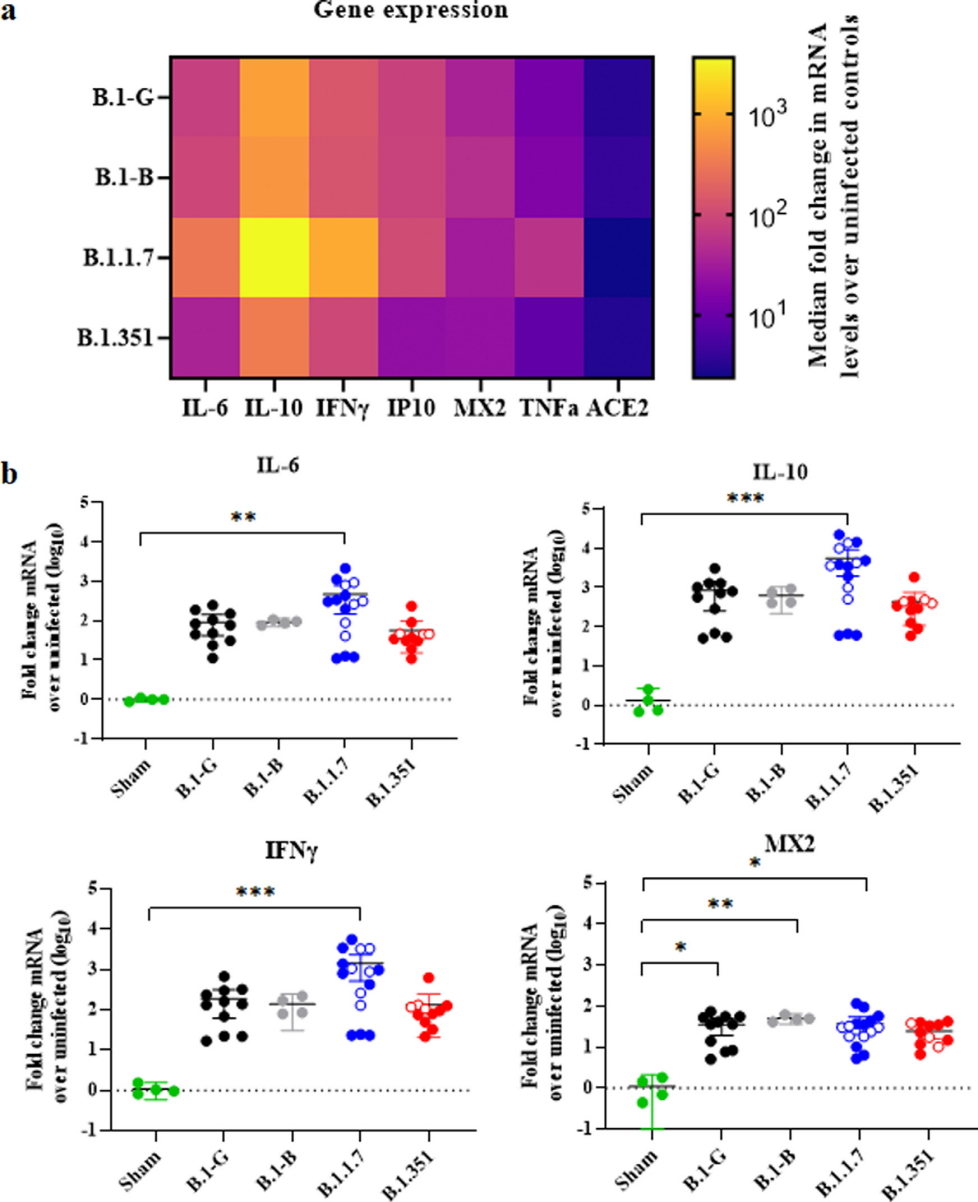
**Expression profiles of selected antiviral, pro-inflammatory, and cytokine genes in the lungs after infection with different SARS-CoV-2 variants**. (a) Heat map showing differential expression of selected antiviral, pro-inflammatory, and cytokine genes in the lungs after infection with the different SARS-CoV-2 variants relative to non-infected control hamsters. (b) RNA levels for IL-6, IL-10, IFN-ɣ and MX2 were determined by RT-qPCR on lung extracts from hamsters infected with B.1-G (n = 11), B.1-B (n = 4), B.1.1.7 (n = 15) or B.1.351 (n = 11) SARS-CoV-2 variants on day 4 post-infection, normalized for β-actin mRNA levels, and fold changes over the median of uninfected controls were calculated using the 2^(−ΔΔCq)^ method. Data presented as fold change over non-infected control. Closed circles represent hamsters infected with SARS-CoV-2 inoculum of 10^5^ TCID50 for all variants, whereas open circles represent 10^4^ TCID_50_ inoculum and 10^2^ TCID_50_ inoculum for B.1.1.7 and B.1.351 variants, respectively. Mean values with 95% CI are shown and statistical significance between variants was calculated by Kruskal–Wallis with Dunn's post hoc test. *P < 0•05, **P < 0•01, ***P < 0•001. All data are from at least two independent experiments except for the B.1-B group.

To investigate the infectivity and virulence of human-adapted VoC B.1.1.7 and B.1.351 (Fig. S1a, b) in hamsters, 6-8 weeks old female Syrian hamsters [15] were intranasally infected with 50 µL containing approximately 1 × 10^5^ TCID_50_ of either basal variants (B.1-G, B.1-B) or VoC (B.1.1.7, B.1.351) SARS-CoV-2 (Fig. 1a). Prototypic strains B.1-G [15] and B.1-B [25] from early 2020 were included as comparators, the latter containing a spike D614G substitution found in early European variants and linked to more efficient transmission [31,32]. At day four post-infection (4 dpi), SARS-CoV-2 replication (Fig. 1b, c), pathology (Fig. 2, Fig. 3), and cytokine expression levels were determined in the lung tissue (Fig. 4 and S4).

Viral RNA loads in the range of 10^7^ RNA copies/mg of lung tissue were consistently detected at 4 dpi for all four strains (Fig. 1b, Table S1). Infectious virus titers were around 10^4^ TCID_50_/mg of lung tissue for all four strains, with no significant differences between them (p = 0·3045, Kruskal-Wallis; Fig. 1c, Table S1); including for the prototypic 2020 strains, i.e. the D614G containing B.1-B strain and the most basal B.1-G strain (p = 0·4753, Kruskal-Wallis), as reported before [31]. Likewise, when B.1-G, B.1.1.7 and B.1.351 were administered at a lower dose of 10^4^ TCID_50_ or 10^2^ TCID_50_ (for B.1.351 only) similar levels of infectious virus titers were detected as with the inoculum of 10^5^ TCID_50_ (Fig. S2), in line with the high susceptibility of hamsters for clinical SARS-CoV-2 isolates [20,33]. As documented before for the B.1-G strain in young female hamsters, [15,16] no overt signs of disease or distress were observed throughout the study, and in general, none of the hamsters showed a marked weight loss (i.e. more than 7%) in any of the infected groups (p = 0·1280, Kruskal-Wallis; Fig. S3, Table S1). Taken together, these results clearly show that VoC B.1.1.7 and B1.351 are able to replicate efficiently in the lower respiratory tract in Syrian hamsters and this to a comparable extent as strains of basal variants.

Basal variants and VoC SARS-CoV-2-induced lung pathology was assessed by microcomputed tomography (micro-CT) at 4 dpi immediately prior to euthanasia (Fig. 2, Table S1) and by post-mortem histopathological analysis (Fig.3, Table S2). Both semi-quantitative scores and quantitative biomarkers derived from micro-CT thorax scans show similar lung disease burden [pulmonary infiltrates (p = 0·8272, Kruskal-Wallis) and bronchial dilation (p = 0·2287, Kruskal-Wallis)] [16] between the hamsters infected with the different SARS-CoV-2 variants (Fig. 2b, c, Table S1).

Hematoxylin/eosin (H&E)-stained images of lungs of hamsters infected with the four variants revealed similar pathological signs with peri-bronchial inflammation and bronchopneumonia in the surrounding alveoli (Fig. 3a). The cumulative histopathological lung scores were 6- to 8-fold higher than the baseline score in untreated, non-infected hamsters (median score of 1·25), with no significant differences between the strains (p = 0·2264, Kruskal-Wallis; Fig. 3b, Table S2). Taken together, both micro-CT and histopathology reveal that the VoC's induce lung pathology in the hamsters to a similar extent as the basal variants do, with the same signs of bronchopneumonia and peribronchial inflammation as previously reported [15,16].

Additionally, the expression levels of a panel of cytokines linked to COVID-19 in humans [34] were measured in the lung tissue of infected hamsters (Fig. 4, Supplementary Table S3). Infection with either of the four strains resulted in an up-regulation of IL-6, IL-10, IFN-λ, IFN-γ, IP-10, MX-2, and TNF-α expression in the range of 10- to 1000-fold compared to non-infected hamsters (Fig. 4, Fig. S4, Supplementary Table S3). Notably, IL-6, IL-10, IFN-γ and TNF-α but not MX-2 expressions were most pronouncedly up-regulated in the B.1.1.7 VoC-infected group as compared to the three other strains, with a statistically significant increase compared to sham (P = 0·001 for IL-6, P = 0·0005 for IL-10, P = 0·0005 for IFN-γ, P = 0·0008 for TNF-α, Kruskal-Wallis test) (Fig. 4b, Fig. S4, Supplementary Table S3). Similar to lung infectious viral loads (Fig. S2), cytokine expression levels were hardly affected by lowering the inoculated virus dose (Fig. 4b and Fig. S4; open circles), arguing for readily saturated conditions. ACE2 receptor expression levels [35] remained rather unaffected and similar between all four strains (p = 0·2188, Kruskal-Wallis; Fig. S4, Supplementary Table S3). If at all, a modest up-regulation appeared to occur in lungs of hamsters infected with the basal B.1-G (p = 0·0144, Kruskal-Wallis) and B.1-B (p = 0·0047, Kruskal-Wallis) variants, yet not with the VoC's. Thus at least at this level, this does not explain differences in infectivity or virulence as observed or suspected in humans.

## 4 Discussion

SARS-CoV-2 genetic diversification was initially considered slow as the virus was spreading in the first months around the globe [36]. However, recently more and more variants are emerging and start dominating regional epidemics in widespread populations [37]. We set out to study the infection and virulence of two prototypic VoC B.1.1.7 and B.1.351 (local isolates from the UK and the South African variants) in the Syrian hamster model using original low passage clinical isolates.

All clinical SARS-CoV-2 isolates studied here, including the VoC B.1.1.7 and B.1.351, replicated efficiently and consistently to high viral loads in hamster lungs (Fig. 1b, c), causing a pathology that resembles bronchopneumonia in COVID patients. However, despite uniformly high viral replication, some variation in disease severity was observed as scored by micro-CT imaging (Fig. 2), histopathology scoring (Fig. 3, Table S2), and cytokine expression profiles (Fig. 4 and Fig. S4) both (*i*) between study groups as well as (*ii*) among individual animals within particular study groups. At first glance, the hamster model may lack sensitivity to discriminate differences in the replication fitness of SARS-CoV-2 from different variants, in particular when using direct intranasal infection with high-titred inocula [31]. Further subtle differences may have been obscured by a lack of statistical power because of the fairly low number of animals per experimental group (n = 4-11), despite well-established precedent [19,20]. However, we [15] and others [27,38] have demonstrated before that strain-specific differences may translate into readily detectable changes in pathology in the hamster model. Of note, the particular virus strains used in former proof-of-principle studies contained tissue culture-adaptive mutations (deletions at the S1/S2 junction close to the furin cleavage site) [15,27,38]. By contrast, when low-passage clinical isolates are used for both the basal and VoC variants (as is the case in the current study), marked quantitative differences cannot be observed. Of note, direct sequencing of viral RNA isolated from infected lungs did not reveal any further sequence evolution in the spike gene during the 4 day course of infection; if at all, a purifying selection and loss of S1/S2 junction variants present in the B.1.351 inoculum.

Nevertheless, our overall observations are fully in line with and hence replicate two key findings from human clinical experience. Firstly, despite some ongoing debate, [7] there seem to be no obvious differences in virulence associated with recently emerging VoC's *versus* earlier basal SARS-CoV-2 variants; definitely, no convergent evolution towards a markedly higher virulence approaching or comparable to what is the case for the highly pathogenic SARS-CoV-1 and MERS coronaviruses [39]. Secondly, also in humans, SARS-CoV-2 infection shows a high variability in disease severity, clinical presentations and outcome in individual patients suffering from COVID-19 [34]. Some of this variability observed in humans may be mirrored in the range and quantitative fluctuation of disease parameters as measured in the hamster model (Fig. 2-4, Fig. S3 and S4). In our study we focused on a single time point for analysis (4 dpi), previously established to represent a peak of B.1-G virus replication [15,16]. Obviously, also others reported significant time-, dose-dependent and inter-individual variation in virus kinetics and pathology [20,31,33]. In conclusion, as far as can be judged from our thorough multi-parameter analysis in Syrian hamsters, VoC B.1.1.7 and B.1.351 compare well in their infectivity and virulence to earlier basal SARS-CoV-2 isolates. It is tempting to speculate that a similar analysis of VoC in alternative models, for instance in ferrets, [18] or likewise in Syrian hamsters of another sex, age [40] or overall health status, or in other hamsters species that present with a wider spectrum of disease manifestations [41] may allow to reveal more subtle differences.

Despite growing insight, it is not known what drives the evolution of SARS-CoV-2. The emergence of future VoC may be driven by any of the following factors: by random selection (founder effects), fitness at the population level (favoring transmission), or viral escape under host immune pressure (antigenic drift), or development of drug resistance under future antiviral therapy. Whatever the cause, as a consequence, any upcoming VoC may spark future COVID-19 epidemics. In an urgent need to characterize current VoC and in anticipation of future needs, the robust hamster model described here will allow to preclinically assess (*i*) virus transmission, (*ii*) vaccine efficacy, and (*iii*) evaluation of pharmacological interventions that target B.1.1.7 and B.1.351 as well as expected future VoC. Importantly, our primary analysis of infection with B.1.1.7 (UK) and B.1.351 (South African) SARS-CoV-2 VoC in hamsters does not reveal evidence for a largely altered phenotype confined to these variants.

## Contributors

All authors read and approved the final version of the manuscript.

R.A., C.S.F., S.J.F.K., R.B., L.D., K.D. and J.N. Conceptualization; R.A., R.B., L.S.F., S.J.F.K., X.Z. and L.S. Methodology; R.A., R.B., L.S., C.S.F., L.S.F, G.V.V. and B.W. Formal analysis and data curation; R.A., R.B. and L.S.F. and L.S. Visualization; K.D., C.S.F., R.B. and R.A. Writing - review & editing.; J.N., P.M. and G.V.V. Resources; R.A., L.D., S.J.F.K., G.V.V., K.D. and J.N. Supervision and project administration; J.N. and K.D. Funding acquisition. R.A., R.B., G.V.V., K.D. and J.N. have verified the underlying data.

## Data sharing statement

All of the data generated or analyzed during this study are included in this published article. All data supporting the findings in this study are also available from the corresponding author upon request.

## Declaration of Competing Interest

Authors declare no conflict of interests.

## Appendix Supplementary materials

Image, application 1

Image, application 2

Image, application 3

Image, application 4
