# Supplementary material for: Comparing infectivity and virulence of emerging SARS-CoV-2 variants in Syrian hamsters
Source: eBioMedicine. 2021 May 25;68:103403. doi: 10.1016/j.ebiom.2021.103403 (PMC8143995; doi:10.1016/j.ebiom.2021.103403)
Supplement: Supplementary file 1 [file mmc1.docx]

**
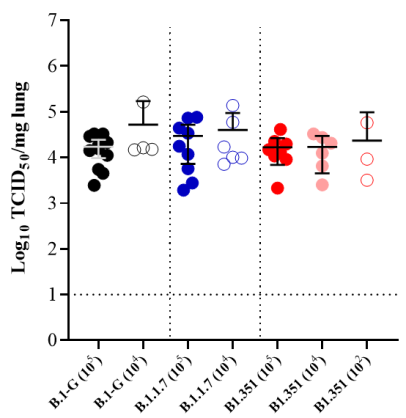
**

**Fig. S1. Genotype and plaque phenotype of different SARS-CoV-2 variants.**

(a) Graphical representation for the SARS-CoV-2 spike gene showing the genotypic difference between the B.1-G, B.1-B, B.1.1.7 and B.1.351 SARS-CoV-2 variants. (b) Plaque phenotype of B.1-G, B.1-B, B.1.1.7 and B.1.351 SARS-CoV-2 variants in Vero E6 cells.

**
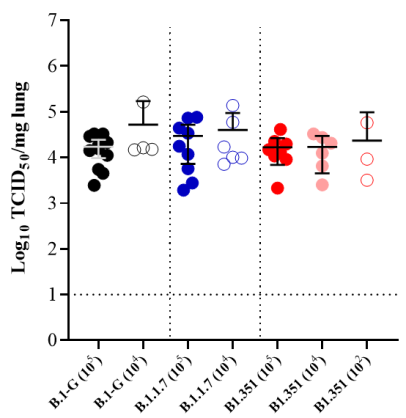
**

**Fig. S2. Infection of Syrian hamsters with different inocula of SARS-CoV-2 variants.** Infectious viral loads in the lungs of hamsters infected with 10^5^ or 10^4^ TCID_50_ of B.1-G (n=11 and 4, respectively), 10^5^ or 10^4^ TCID_50_ of B.1.1.7 (n=9 and 6, respectively) or 10^5^, 10^4^ or 10^2^ TCID_50_ of B.1.351 (n=8, 6 and 3, respectively) SARS-CoV-2 variants on day 4 post-infection (pi) are expressed as log_10_ SARS-CoV-2 RNA copies per mg lung tissue. Individual data and mean values with 95% CI are presented.

**
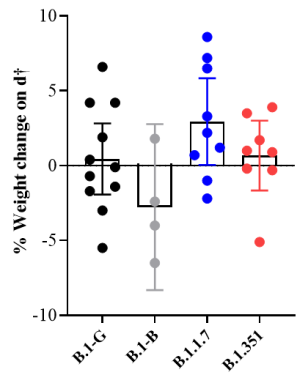
**

**Fig. S3. Weight change in hamsters infected with different SARS-CoV-2 variants.** Weight change at day 4 post-infection with 10^5^ TCID_50_ of B.1-G (n=11), B.1-B (n=4), B.1.1.7 (n=9) or B.1.351 (n=8) SARS-CoV-2 variants represented in percentage and normalized to the body weight at the time of infection. Mean values with 95% CI are presented. Data were analyzed by Kruskal-Wallis with Dunn’s post hoc test. All data are from at least two independent experiments except for the B.1-B group.


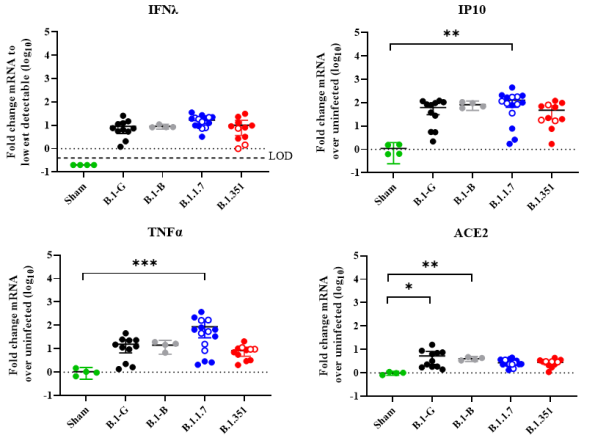


Fig. S4. Expression profiles of selected antiviral, pro-inflammatory, and cytokine genes in the lungs after infection with different SARS-CoV-2 variants. RNA levels for each marker gene were determined by RT-qPCR on lung extracts from hamsters infected with B.1-G (n=11), B.1-B (n=4), B.1.1.7 (n=15) or B.1.351 (n=11) SARS-CoV-2 variants on day 4 post-infection, normalized for β-actin mRNA levels, and fold changes over the median of uninfected controls were calculated using the 2^(−ΔΔCq)^ method. Only for IFN-λ, where all uninfected control animals had undetectable RNA levels, fold changes were calculated over the lowest detectable value. Data presented as fold change over non-infected control. Closed circles represent hamsters infected with SARS-CoV-2 inoculum of 10^5^ TCID_50_ for all variants, whereas open circles represent 10^4^ TCID_50_ inoculum and 10^2^ TCID_50_ inoculum for B.1.1.7 and B.1.351 variants, respectively. Mean values with 95% CI are presented and statistical significance between variants was calculated by Kruskal–Wallis with Dunn’s post hoc test. *P < 0·05, **P < 0·01, ***P < 0·001. All data are from at least two independent experiments except for the B.1-B group.
