# Supplementary material for: Comparing infectivity and virulence of emerging SARS-CoV-2 variants in Syrian hamsters
Source: eBioMedicine. 2021 May 25;68:103403. doi: 10.1016/j.ebiom.2021.103403 (PMC8143995; doi:10.1016/j.ebiom.2021.103403)
Supplement: Supplementary file 2 [file mmc2.docx]

**Supplementary table S1: Raw data of Figures 1-3 and Supplementary Figure S2**

|  | **Hamster ID** | **RNA copies/mg lung** | **TCID50/mg lung** | **Weight at infection (g)** | **% weight change on D4** | **Histology** | **CT score lung** | **CT score bronchi** | **ALV (%)** | **NALV (%)** |
| --- | --- | --- | --- | --- | --- | --- | --- | --- | --- | --- |
| **B.1-G** | 1 | 1.54E+07 | 2.46E+03 | 100.7 | -1.4 | 10 | 1.25 | 0.25 | poor resolution | poor resolution |
|  | 2 | 3.47E+07 | 5.47E+03 | 80.6 | 4.2 | 11 | 6 | 3.5 | 85.9 | 14.1 |
|  | 3 | 1.03E+07 | 2.13E+04 | 97.9 | -5.5 | 9 | 0.5 | 2.25 | 91.4 | 8.6 |
|  | 4 | 4.59E+07 | 1.84E+04 | 96.4 | -0.1 | 8 | 1 | 1 | 89.2 | 10.8 |
|  | 5 | 1.57E+07 | 1.56E+04 | 92.1 | 4.2 | 11 | 2.75 | 2.5 | 90.0 | 10.0 |
|  | 6 | 9.19E+05 | 1.11E+04 | 89.8 | 6.6 | 9 | 3.5 | 1 | 82.3 | 17.7 |
|  | 7 | 1.50E+06 | 3.28E+04 | 105.6 | 0.4 | 9 | 6 | 2.5 | 65.8 | 34.2 |
|  | 8 | 5.97E+06 | 1.42E+04 | 110.8 | -1.7 | 6 | 6.25 | 2.5 | 57.4 | 42.6 |
|  | 9 | 1.11E+07 | 4.47E+03 | 87.5 | -3.0 | 8 | 7 | 0.75 | 68.5 | 31.5 |
|  | 10 | 3.62E+07 | 2.91E+04 | 85.8 | -0.7 | 8 | 5.25 | 3.25 | 74.6 | 25.4 |
|  | 11 | 2.25E+07 | 3.26E+04 | 89.9 | 1.9 | 7 | 1.75 | 3 | 88.8 | 11.2 |
| mean [95% CI] |  | 1.82E+07 [8.15E+06 ; 2.82E+07] | 1.70E+04 [9.67E+03 ; 2.44E+04] | 94.28 [88.24 ; 100.3] | 0.45 [-1.93 ; 2.82] | 8.73 [7.68 ; 9.77] | 3.75 [2.12 ; 5.38] | 2.05 [1.30 ; 2.79] | 79.39 [70.79 ; 87.99] | 20.61 [12.01 ; 29.21] |
| **B.1-B** | 12 | 3.50E+07 | 3.94E+04 | 113.6 | -2.4 | 7 | 3.75 | 0.5 | 79.6 | 20.4 |
|  | 13 | 3.27E+07 | 1.98E+05 | 108.2 | -4.0 | 6 | 0.25 | 2 | 94.8 | 5.2 |
|  | 14 | 3.06E+07 | 3.51E+04 | 106.4 | -6.5 | 8 | 6.25 | 2.25 | 83.0 | 17.0 |
|  | 15 | 6.28E+07 | 1.20E+04 | 106.9 | 1.8 | 7 | 2 | 2.25 | 90.5 | 9.5 |
| mean [95% CI] |  | 4.03E+07 [1.62E+07 ; 6.43E+07] | 7.11E+04 [-6.48E+04 ; 2.07E+05] | 108.8 [103.5 ; 114.0] | -2.78 [-8.32 ; 2.77] | 7.00 [5.70 ; 8.30] | 3.06 [-1.01 ; 7.14] | 1.75 [0.41 ; 3.09] | 87.01 [76.01 ; 98.00] | 12.99 [2.00 ; 23.99] |
| **B.1.1.7** | 16 | 4.01E+07 | 7.59E+04 | 91.5 | 0.7 | 9 | 0 | 0 | 86.3 | 13.7 |
|  | 17 | 4.57E+07 | 7.24E+04 | 92.3 | 2.2 | 9 | 0.25 | 2.75 | 90.9 | 9.1 |
|  | 18 | 7.43E+06 | 1.76E+04 | 85.1 | 1.2 | 5 | 6.5 | 3 | 74.3 | 25.7 |
|  | 19 | 1.35E+07 | 4.38E+04 | 98.5 | 7.2 | 7.5 | 0 | 2.5 | 93.3 | 6.7 |
|  | 20 | 8.14E+06 | 1.17E+04 | 90.7 | 6.5 | 7 | 6.5 | 3 | 74.5 | 25.5 |
|  | 21 | 1.37E+07 | 5.66E+03 | 87.2 | 3.3 | 5 | 1 | 2.5 | 89.6 | 10.4 |
|  | 22 | 1.76E+08 | 2.77E+03 | 80.4 | -1.0 | 6 | 6 | 0.75 | 76.3 | 23.7 |
|  | 23 | 1.76E+08 | 1.93E+03 | 84.6 | -2.2 | 11 | 6.75 | 2.75 | 74.3 | 25.7 |
|  | 24 | 4.40E+08 | 3.39E+04 | 93.6 | 8 | 9.5 | 0 | 3.25 | 92.8 | 7.2 |
| mean [95% CI] |  | 1.02E+08 [-8.30E+06 ; 2.12E+08] | 2.95E+04 [7.24E+03 ; 5.18E+04] | 89.32 [85.09 ; 93.55] | 2.88 [0.07 ; 5.69] | 7.67 [6.05 ; 9.29] | 3.00 [0.48 ; 5.52] | 2.28 [1.42 ; 3.14] | 83.60 [77.03 ; 90.17] | 16.40 [9.83 ; 22.97] |
| **B.1.351** | 25 | 4.95E+06 | 2.13E+03 | 94.8 | 3.5 | 9.5 | 6 | 0 | 73.7 | 26.3 |
|  | 26 | 1.21E+07 | 2.24E+04 | 91.7 | 0.9 | 6 | 2 | 3 | 88.0 | 12.0 |
|  | 27 | 1.64E+06 | 1.32E+04 | 89.2 | -0.2 | 11 | 7.25 | 2.5 | 72.9 | 27.1 |
|  | 28 | 1.23E+07 | 1.48E+04 | 93.0 | 3.9 | 6.5 | 0.5 | 2.75 | 93.4 | 6.6 |
|  | 29 | 2.16E+07 | 1.97E+04 | 84.0 | -5.1 | 10.5 | 5.75 | 3.75 | 80.6 | 19.3 |
|  | 30 | 2.93E+08 | 1.07E+04 | 83.6 | 1.7 | 10.5 | 5.25 | 3.5 | 78.7 | 21.3 |
|  | 31 | 1.25E+08 | 9.07E+03 | 88.7 | 1 | 8.5 | 2 | 2.5 | 81.5 | 18.5 |
|  | 32 | 5.13E+08 | 4.10E+04 | 91.6 | -0.3 | 8 | bad scan | bad scan | bad scan | bad scan |
| mean [95% CI] |  | 1.23E+08 [-3.32E+07 ; 2.79E+08] | 1.66E+04 [6.86E+03 ; 2.64E+04] | 89.58 [86.18 ; 92.97] | 0.68 [-1.66 ; 3.01] | 8.81 [7.23 ; 10.39] | 4.11 [1.74 ; 6.48] | 2.57 [1.43 ; 3.71] | 81.27 [74.46 ; 88.09] | 18.72 [11.91 ; 25.54] |
