# Supplementary material for: Comparing infectivity and virulence of emerging SARS-CoV-2 variants in Syrian hamsters
Source: eBioMedicine. 2021 May 25;68:103403. doi: 10.1016/j.ebiom.2021.103403 (PMC8143995; doi:10.1016/j.ebiom.2021.103403)
Supplement: Supplementary file 3 [file mmc3.docx]

**Supplementary table S2: Detailed histopathology scoring for hamsters infected with different SARS-CoV-2 variants**

| **Group** | **hamster ID** | **congestion** | **intralveolar hemorrhage** | **lymphoid follicles** | **apoptotic bodies in bronchus wall** | **necrotizing bronchiolitis** | **perivascular edema** | **bronchopneumonia** | **% involved** | **perivascular inflammation** | **peribronchial inflammation** | **vaculitis** | **endothelialitis** | **perivascular cuff** | **intraluminal PMN** | **mesothelial hyperplasia** | **mesothelial inflammation** | **mesothelial fibrosis** | **intraalveolar edema** | **Score** |
| --- | --- | --- | --- | --- | --- | --- | --- | --- | --- | --- | --- | --- | --- | --- | --- | --- | --- | --- | --- | --- |
| **B.1-G** | 1 | 1 |  |  | 1 |  | 1 | 2 | 20 | 2 | 2 | 1 | 1 | 2 | 1 | 0 | 0.5 | 1 | 0 | 10 |
|  | 2 | 1 | 1 |  | 1 |  | 1 | 2 | 30 | 2 | 2 | 1 | 1 | 1 | 0 | 1 | 0 | 0 | 1 | 11 |
|  | 3 | 1 |  |  | 1 |  | 1 | 1 | 5 | 2 | 2 | 1 | 1 | 1 | 1 | 0.5 | 0 | 1 | 0 | 9 |
|  | 4 | 1 |  |  | 1 |  |  | 1 | 5 | 2 | 2 | 1 | 1 | 2 | 1 | 1 | 0.5 | 0 | 0 | 8 |
|  | 5 | 1 | 2 |  | 1 |  |  | 3 | 50 | 1 | 2 | 1 | 1 | 1 | 1 | 1 | 0 | 0 | 1 | 11 |
|  | 6 | 1 | 1 |  | 1 |  |  | 2 | 20 | 1 | 2 | 1 | 1 | 1 | 0 | 1 | 0 | 0 | 0 | 9 |
|  | 7 | 1 | 2 | 2 | 1 |  |  | 2 | 20 | 1 | 1 | 1 | 1 | 2 | 0 | 0 | 0.5 | 1 | 1 | 9 |
|  | 8 | 1 | 1 |  | 1 |  |  | 1 | 5 |  | 1 | 1 | 1 | 0 | 0 | 0 | 0 | 0 | 0 | 6 |
|  | 9 | 1 |  |  | 1 |  |  | 2 | 30 | 1 | 2 | 1 | 1 | 2 | 1 | 1 | 0 | 0 | 1 | 8 |
|  | 10 | 1 |  |  | 1 |  |  | 2 | 10 | 1 | 2 | 1 | 1 | 2 | 1 | 1 | 0 | 0 | 0 | 8 |
|  | 11 | 1 |  |  | 1 |  |  | 1 | 5 | 1 | 2 | 1 | 1 | 1 | 1 | 1 | 0 | 0 | 0 | 7 |
| **B.1-B** | 12 | 1 | 1 |  | 1 |  |  | 1 | 5 | 1 | 1 | 1 | 1 | 1 | 1 | 0 | 0 | 1 | 0 | 7 |
|  | 13 | 1 |  |  | 1 |  |  | 1 | 5 | 1 | 1 | 1 | 1 | 1 | 1 | 0 | 0 | 1 | 0 | 6 |
|  | 14 | 1 | 1 |  | 1 |  | 1 | 1 | 5 | 1 | 1 | 1 | 1 | 1 | 1 | 0 | 0 | 0 | 0 | 8 |
|  | 15 | 1 |  | 1 | 1 |  | 1 | 1 | 5 | 2 | 1 |  | 1 | 2 | 1 | 1 | 1 | 1 | 0 | 7 |
| **B.1.1.7** | 16 | 1 |  |  | 1 |  |  | 2 | 10 | 2 | 2 | 1 | 1 | 2 | 1 | 1 | 0 | 0 | 1 | 9 |
|  | 17 | 1 |  |  | 1 |  |  | 2 | 30 | 2 | 2 | 1 | 1 | 2 | 1 | 1 | 0 | 0 | 1 | 9 |
|  | 18 | 1 |  |  | 1 |  |  | 1 | 5 | 1 | 1 |  | 1 | 1 | 0 | 1 | 0 | 1 | 0 | 5 |
|  | 19 | 1 |  |  | 1 |  |  | 2 | 30 | 2 | 1 | 0.5 | 1 | 2 | 0 | 1 | 0 | 1 | 1 | 7.5 |
|  | 20 | 1 |  |  | 1 |  |  | 2 | 30 | 2 | 1 |  | 1 | 1 | 0 | 1 | 1 | 0 | 1 | 7 |
|  | 21 | 1 |  |  | 1 |  |  | 1 | 5 | 1 | 1 |  | 1 | 1 | 0 | 0 | 0 | 0 | 0 | 5 |
|  | 22 | 1 | 1 |  | 1 |  |  | 1 | 10 | 1 | 1 |  | 1 | 1 | 1 | 0 | 0 | 0 | 0 | 6 |
|  | 23 | 1 | 1 |  | 1 |  | 1 | 3 | 60 | 2 | 1 | 1 | 1 | 3 | 1 | 1 | 1 | 1 | 1 | 11 |
|  | 24 | 1 | 1 |  | 1 |  |  | 2 | 20 | 2 | 2 | 0.5 | 1 | 2 | 1 | 1 | 1 | 0 | 0 | 9.5 |
| **B.1.351** | 25 | 1 |  |  | 1 |  | 1 | 3 | 50 | 2 | 1 | 0.5 | 1 | 2 | 0 | 1 | 1 | 0 | 0 | 9.5 |
|  | 26 | 1 |  |  | 1 |  |  | 2 | 30 | 1 | 1 |  | 1 | 1 | 1 | 1 | 1 | 0 | 0 | 6 |
|  | 27 | 1 | 1 |  | 1 |  | 1 | 3 | 60 | 2 | 1 | 1 | 1 | 2 | 0 | 1 | 1 | 0 | 1 | 11 |
|  | 28 | 1 |  |  | 1 |  |  | 1 | 5 | 1 | 2 | 0.5 | 1 | 1 | 1 | 1 | 0 | 0 | 1 | 6.5 |
|  | 29 | 1 | 1 |  | 1 |  | 1 | 3 | 60 | 1 | 2 | 0.5 | 1 | 2 | 0 | 1 | 0 | 0 | 1 | 10.5 |
|  | 30 | 1 | 1 |  | 1 |  | 1 | 2 | 20 | 2 | 2 | 0.5 | 1 | 2 | 0 | 1 | 0 | 0 | 1 | 10.5 |
|  | 31 | 1 |  |  | 1 |  |  | 2 | 20 | 2 | 2 | 0.5 | 1 | 2 | 1 | 1 | 1 | 0 | 1 | 8.5 |
|  | 32 | 1 | 1 |  | 1 |  |  | 2 | 20 | 1 | 2 |  | 1 | 1 | 0 | 1 | 0 | 1 | 0 | 8 |
