# Supplementary material for: Comparing infectivity and virulence of emerging SARS-CoV-2 variants in Syrian hamsters
Source: eBioMedicine. 2021 May 25;68:103403. doi: 10.1016/j.ebiom.2021.103403 (PMC8143995; doi:10.1016/j.ebiom.2021.103403)
Supplement: Supplementary file 4 [file mmc4.docx]

**Supplementary table S3: Raw data of Figure 4 and Supplementary Figure S4**

|  | **Hamster ID** | **Fold change over median of sham or fold change over lowest detectable (for IFNλ) (2^-ΔΔCq method)** | | | | | | | |
| --- | --- | --- | --- | --- | --- | --- | --- | --- | --- |
|  |  | **IL-6** | **IL-10** | **IFNλ** | **IFNγ** | **IP10** | **MX2** | **TNFα** | **ACE2** |
| **B.1-G** | 1 | 48.17 | 564.18 | 2.07 | 138.62 | 38.45 | 36.25 | 12.73 | 7.16 |
|  | 2 | 105.42 | 803.41 | 7.41 | 163.71 | 105.79 | 56.10 | 15.03 | 2.31 |
|  | 3 | 247.28 | 3061.45 | 1.21 | 668.60 | 79.07 | 56.10 | 45.89 | 1.84 |
|  | 4 | 44.94 | 724.08 | 6.54 | 132.97 | 122.36 | 74.03 | 9.51 | 3.23 |
|  | 5 | 76.64 | 286.03 | 14.93 | 68.83 | 97.34 | 41.36 | 11.47 | 16.00 |
|  | 6 | 83.87 | 1016.93 | 5.39 | 236.39 | 27.95 | 14.03 | 23.43 | 7.52 |
|  | 7 | 187.40 | 1287.18 | 11.16 | 320.68 | 84.16 | 36.00 | 22.78 | 6.32 |
|  | 8 | 148.06 | 1287.18 | 25.81 | 301.29 | 116.57 | 52.71 | 25.11 | 8.82 |
|  | 9 | 23.93 | 54.53 | 12.44 | 22.21 | 5.44 | 7.72 | 1.36 | 1.73 |
|  | 10 | 31.10 | 68.46 | 7.60 | 21.95 | 5.61 | 8.36 | 2.25 | 1.41 |
|  | 11 | 11.33 | 51.14 | 5.68 | 16.91 | 2.21 | 5.10 | 1.64 | 1.88 |
| mean [95% CI] |  | 91.65 [41.46 ; 141.84] | 836.78 [251.18 ; 1422.38] | 9.11 [4.47 ; 13.75] | 190.20 [61.27 ; 319.12] | 62.27 [30.59 ; 93.95] | 35.25 [19.41 ; 51.09] | 15.56 [6.65 ; 24.47] | 5.29 [2.28 ; 8.31] |
| **B.1-B** | 12 | 96.34 | 754.83 | 8.34 | 169.48 | 96.00 | 49.87 | 16.56 | 3.68 |
|  | 13 | 87.43 | 398.93 | 8.28 | 85.33 | 69.31 | 47.84 | 14.72 | 3.41 |
|  | 14 | 75.06 | 421.68 | 8.46 | 80.17 | 58.69 | 41.64 | 6.82 | 4.35 |
|  | 15 | 106.15 | 922.88 | 11.08 | 220.56 | 106.52 | 62.25 | 19.29 | 4.76 |
| mean [95% CI] |  | 91.25 [70.20 ; 112.29] | 624.58 [215.75 ; 1033.41] | 9.04 [6.87 ; 11.21] | 138.89 [30.49 ; 247.29] | 82.63 [47.05 ; 118.21] | 50.40 [36.65 ; 64.15] | 14.35 [5.82 ; 22.87] | 4.05 [3.07 ; 5.03] |
| **B.1.1.7** | 16 | 195.36 | 1910.85 | 35.75 | 429.05 | 200.16 | 95.01 | 33.59 | 4.00 |
|  | 17 | 433.53 | 4870.99 | 22.63 | 1384.36 | 169.48 | 57.28 | 81.01 | 2.36 |
|  | 18 | 302.33 | 3420.52 | 12.73 | 800.63 | 102.18 | 37.27 | 65.34 | 3.71 |
|  | 19 | 1120.56 | 14362.31 | 20.11 | 3456.27 | 210.11 | 44.32 | 216.77 | 2.35 |
|  | 20 | 2120.22 | 22226.61 | 27.47 | 5575.94 | 450.38 | 116.97 | 372.22 | 3.34 |
|  | 21 | 337.79 | 3821.70 | 9.92 | 978.89 | 79.07 | 34.78 | 65.34 | 4.50 |
|  | 22 | 12.73 | 60.92 | 19.21 | 23.19 | 7.77 | 10.14 | 2.08 | 1.32 |
|  | 23 | 10.96 | 61.27 | 9.35 | 23.35 | 2.63 | 5.30 | 2.63 | 2.38 |
|  | 24 | 12.00 | 66.71 | 3.24 | 24.73 | 1.73 | 6.42 | 2.90 | 2.32 |
| **B.1.1.7 10^4^ TCID_50_** | 33 | 261.38 | 3590.58 | 20.82 | 882.22 | 116.57 | 18.38 | 45.89 | 2.13 |
|  | 34 | 922.88 | 13493.72 | 18.51 | 3338.54 | 180.39 | 30.27 | 163.14 | 1.54 |
|  | 35 | 797.86 | 9946.68 | 22.01 | 3247.24 | 174.25 | 30.27 | 168.90 | 2.71 |
|  | 36 | 315.17 | 4240.45 | 14.32 | 1063.79 | 86.52 | 23.92 | 54.95 | 2.38 |
|  | 37 | 40.79 | 501.46 | 7.89 | 130.24 | 36.13 | 18.38 | 8.40 | 3.56 |
|  | 38 | 88.65 | 996.00 | 7.36 | 260.47 | 94.03 | 32.67 | 15.67 | 2.33 |
| mean [95% CI] |  | 464.81 [147.11 ; 782.51] | 5571.38 [1941.44 ; 9201.33] | 16.75 [11.96 ; 21.55] | 1441.26 [513.84 ; 2368.68] | 127.43 [64.10 ; 190.76] | 37.43 [20.00 ; 54.85] | 86.59 [29.28 ; 143.89] | 2.73 [2.23 ; 3.23] |
| **B.1.351** | 25 | 31.34 | 294.07 | 2.71 | 75.85 | 19.77 | 14.93 | 8.75 | 3.01 |
|  | 26 | 36.76 | 340.14 | 8.11 | 94.68 | 70.28 | 33.13 | 7.11 | 2.30 |
|  | 27 | 232.32 | 1820.35 | 9.19 | 623.83 | 64.22 | 34.78 | 20.82 | 3.61 |
|  | 28 | 90.51 | 261.38 | 13.18 | 79.62 | 120.68 | 39.95 | 5.62 | 3.43 |
|  | 29 | 34.54 | 455.09 | 23.26 | 126.68 | 98.70 | 42.52 | 10.27 | 4.41 |
|  | 30 | 10.87 | 59.21 | 3.23 | 21.70 | 1.70 | 6.71 | 2.04 | 1.72 |
|  | 31 | 30.32 | 126.90 | 31.59 | 50.10 | 22.29 | 23.01 | 3.26 | 1.09 |
|  | 32 | 19.44 | 90.48 | 9.83 | 32.46 | 7.66 | 11.68 | 2.97 | 2.11 |
| **B.1.351 10^2^ TCID_50_** | 39 | 47.18 | 436.55 | 1.00 | 132.97 | 16.85 | 10.13 | 9.58 | 3.03 |
|  | 40 | 44.32 | 396.18 | 7.46 | 106.52 | 80.73 | 38.05 | 9.45 | 2.95 |
|  | 41 | 45.57 | 487.75 | 1.45 | 117.38 | 18.06 | 17.51 | 11.24 | 3.07 |
| mean [95% CI] |  | 56.65 [15.20 ; 98.10] | 433.46 [108.65 ; 758.28] | 10.09 [3.66 ; 16.52] | 132.89 [20.71 ; 245.07] | 47.36 [19.84 ; 74.87] | 24.76 [15.86 ; 33.67] | 8.28 [4.77 ; 11.80] | 2.79 [2.17 ; 3.42] |
| **Sham** | 42 | 0.88 | 0.68 | ND | 0.96 | 0.65 | 0.69 | 1.49 | 0.81 |
|  | 43 | 1.11 | 0.74 | ND | 0.82 | 0.63 | 0.44 | 0.67 | 1.01 |
|  | 44 | 1.00 | 2.55 | ND | 1.04 | 1.54 | 1.44 | 0.96 | 1.10 |
|  | 45 | 1.00 | 1.35 | ND | 1.53 | 1.61 | 1.82 | 1.04 | 0.99 |
| mean [95% CI] |  | 1.00 [0.85 ; 1.15] | 1.33 [-0.05 ; 2.71] | / | 1.09 [0.60 ; 1.58] | 1.11 [0.25 ; 1.97] | 1.10 [0.08 ; 2.12] | 1.04 [0.50 ; 1.58] | 0.98 [0.78 ; 1.17] |
